# Investigating the contributions of circadian pathway and insomnia risk genes to autism and sleep disturbances

**DOI:** 10.1038/s41398-022-02188-2

**Published:** 2022-10-03

**Authors:** Rackeb Tesfaye, Guillaume Huguet, Zoe Schmilovich, Thomas Renne, Mor Absa Loum, Elise Douard, Zohra Saci, Martineau Jean-Louis, Jean Luc Martineau, Rob Whelan, Sylvane Desrivieres, Andreas Heinz, Gunter Schumann, Caroline Hayward, Mayada Elsabbagh, Sebastien Jacquemont

**Affiliations:** 1 McGill University, Neurology and Neurosurgery, Montreal Neurological Institute, Azrieli Centre for Autism Research, Montreal, Canada; 2 UHC Sainte-Justine Research Center, Université de Montréal, Montreal, Canada; 3 McGill University, Human Genetics, Montreal, Canada; 4 Institut National de la Santé et de la Recherche Médicale, INSERM U A10 “Trajectoires développementales en psychiatrie”, Université Paris-Saclay, Paris-Saclay, CNRS, Centre Borelli, Gif-sur-Yvette, France; 5 Global Brain Health Institute and School of Psychology, Trinity College Dublin, Dublin, Ireland; 6 Centre for Population Neuroscience and Precision Medicine (PONS), Institute of Psychiatry, Psychology & Neuroscience, SGDP Centre, King’s College London, London, United Kingdom; 7 Department of Psychiatry and Psychotherapy CCM, Charité – Universitätsmedizin Berlin, corporate member of Freie Universität Berlin, Humboldt-Universität zu Berlin, and Berlin Institute of Health, Berlin, Germany; 8 PONS Research Group, Dept of Psychiatry and Psychotherapy, Campus Charite Mitte, Humboldt University, Berlin and Leibniz Institute for Neurobiology, Magdeburg, Germany, and Institute for Science and Technology of Brain-inspired Intelligence (ISTBI), Fudan University, Shanghai, People’s Republic of China; 9 MRC Human Genetics Unit, Institute of Genetics and Cancer, University of Edinburgh, Western General Hospital, Edinburgh, EH4 2XU United Kingdom; 10 Generation Scotland, Centre for Genomic and Experimental Medicine, Institute of Genetics and Cancer, University of Edinburgh, Western General Hospital, Edinburgh, EH4 2XU United Kingdom

**Keywords:** Clinical genetics, Autism spectrum disorders

## Abstract

Sleep disturbance is prevalent in youth with Autism Spectrum Disorder (ASD). Researchers have posited that circadian dysfunction may contribute to sleep problems or exacerbate ASD symptomatology. However, there is limited genetic evidence of this. It is also unclear how insomnia risk genes identified through GWAS in general populations are related to ASD and common sleep problems like insomnia traits in ASD. We investigated the contribution of copy number variants (CNVs) encompassing circadian pathway genes and insomnia risk genes to ASD risk as well as sleep disturbances in children with ASD. We studied 5860 ASD probands and 2092 unaffected siblings from the Simons Simplex Collection (SSC) and MSSNG database, as well as 7509 individuals from two unselected populations (IMAGEN and Generation Scotland). Sleep duration and insomnia symptoms were parent reported for SSC probands. We identified 335 and 616 rare CNVs encompassing circadian and insomnia risk genes respectively. Deletions and duplications with circadian genes were overrepresented in ASD probands compared to siblings and unselected controls. For insomnia-risk genes, deletions (not duplications) were associated with ASD in both cohorts. Results remained significant after adjusting for cognitive ability. CNVs containing circadian pathway and insomnia risk genes showed a stronger association with ASD, compared to CNVs containing other genes. Circadian genes did not influence sleep duration or insomnia traits in ASD. Insomnia risk genes intolerant to haploinsufficiency increased risk for insomnia when duplicated. CNVs encompassing circadian and insomnia risk genes increase ASD liability with little to no observable impacts on sleep disturbances.

## Introduction

Sleep problems have been reported in 40 to 80% of youth with Autism Spectrum Disorder (ASD) [1–4]. These problems emerge early in infancy and continue throughout the lifespan among those diagnosed with ASD [5]. Sleep is crucial for successful mental and physical well-being, and such disturbances may contribute to exacerbating symptomatology and comorbidities in ASD [6]. The most commonly reported sleep disturbances in ASD include poor sleep duration and insomnia, defined by night awakenings and/or delayed sleep onset that contribute to negative daytime functioning consequences. Recent studies have shown that sleep onset problems are predictive of an ASD diagnoses in high-risk infants [7], while both longer and shorter sleep duration have been longitudinally associated with behavioral regulation issues in children with ASD [8].

Sleep disturbances are commonly reported in monogenic syndromes (e.g., Rett, Angelman, and Phelan McDermid syndromes) with high cases of ASD, suggesting biological processes underlying elevated sleep problems in ASD may be informed by genetic factors [9, 10]. However, genetic factors contributing to sleep problems in idiopathic ASD remains understudied. Genetic factors are major contributors to ASD liability as well as sleep disturbance. Heritability of ASD is between 64 and 90% [11], while the heritability of self-reported sleep duration and insomnia in adults ranges between 0.25–0.44% and 0.22–0.59% respectively [12, 13].

Sleep is regulated by two distinct yet interacting biological processes; (1) sleep-wake homeostasis (“process s”) and (2) the circadian rhythm (“process c”) [14]. A homeostatic sleep drive, corresponding to the biological need for sleep. The daily timing of sleep, in addition to other physiological functions (i.e., behavioral, hormonal, and attentional processes), is regulated by the circadian system [14]. It’s composed of an oscillatory rhythm that fluctuates to an approximate 24 h period. Circadian rhythms generated in the suprachiasmatic nucleus (SCN) are regulated by “core clock” genes through a network of positive and negative feedback loops genes and influenced by environmental cues such as light, temperature, and social activities [14, 15]. Additionally, subsequent peripheral clock genes and circadian pathways not only restricted to the SCN have since been uncovered, going beyond a “core” loop to involve hundreds of genetic modifiers that directly and indirectly interact with core loops to entrain circadian rhythms [11].

Earlier genetic studies of sleep traits tested the hypothesis that mutations in core clock genes would affect circadian sleep phenotypes [13, 16]. For instance, common variants in *CRY1* were associated with delayed sleep phase disorder [17], while variants in *PER2* [18, 19] and *CKIδ* [20] led to advanced sleep phase disorders.

Researchers have put forth a circadian theory of ASD risk, suggesting that circadian dysfunction may underlie elevated sleep problems, which increases the susceptibility of an ASD diagnoses, along with other difficulties related to circadian misalignment (e.g., social cues, attentional processes, etc.) [21–23]. The link between ASD and circadian pathways has been investigated in a few studies, however, the relationship remains unclear. Studies, mainly focusing on core clock genes, were not able to associate common variants in circadian genes with ASD or sleep disturbances in this population [24–27]. As an example, a 2019 study showed SNPs within 25 clock and melatonin genes were not associated with broad night and daytime sleep issues in 2065 ASD youth in the Simon Simplex Collection [26]. The relationship between the circadian system and ASD has also been investigated through melatonin regulation. Atypical levels of melatonin (neurohormone that helps reset the biological clock [28]) have been found in ASD and studies suggest that common genetic variants altering melatonin synthesis are associated with sleep phenotypes in youth with ASD [21, 29, 30]. However, there has been no comprehensive study investigating the role of the larger circadian pathway beyond ‘core clock’ and melatonin synthesis genes.

Further, most of the genetic contribution and biological processes implicated in sleep disturbances lie outside the circadian pathway. In addition to homeostasis regulation, mouse models of monogenic syndromes associated with ASD reveal the presence of sleep disturbance with minimal impact on circadian rhythms [31, 32]. Moreover, recent genome-wide association studies on insomnia identified only a few circadian genes among the over 1000 insomnia risk-genes [33, 34].

In particular, there has been no focus on rare variants implicating circadian and insomnia-risk genes. The importance of rare copy number variants (CNVs; defined as genomic deletions or duplications >1 kb) to ASD is well demonstrated and previous studies have replicated the association between 16 specific recurrent CNVs and ASD [35]. Our team has also shown that rare non-recurrent CNVs distributed across the genome encompassing coding genes intolerant to haploinsufficiency are associated with increased liability to ASD [36]. Whether circadian and insomnia-risk genes encompassed in CNVs contribute to ASD or to common sleep duration and insomnia issues in the population is unknown. In addition, the effects of these CNVs (rather than SNPs) have yet to be investigated in either a general population or ASD population.

We hypothesize that genomic variants disrupting pathways involved in circadian rhythms and insomnia are linked to ASD risk. Our aim was to understand the relationship between autism risk, sleep disturbances, circadian pathways, and insomnia-risk genes.

We investigated circadian pathway and insomnia risk genes disrupted by CNVs in two ASD cohorts (Simons Simplex Collection and MSSNG), and in unaffected siblings (Simons Simplex Collection), and individuals from unselected populations (Generation Scotland and IMAGEN). We also characterized the effect of these CNVs on parent-reported sleep duration and insomnia traits.

## Materials and methods

### Cohorts

#### Autism datasets

The Simons Simplex Collection (SSC) includes 2569 simplex families with one ASD proband per family and 2851 unaffected siblings. The MSSNG dataset was used as an independent replication cohort and includes 3426 probands with ASD from multiplex families [37–39].

#### General population

The general population was pooled from two previously described cohorts. The longitudinal IMAGEN dataset containing 2093 adolescents [40]. The second cohort included 16,916 adults from Generation Scotland: the Scottish Family Health Study (GS) [41]. Only one individual per family was included.

### CNV calling, filtering, and annotation

In all cohorts except MSSNG, SNP array data was available and CNVs were called using Penn CNV and QuantiSNP based on published pipelines [42]. For MSSNG, CNV’s were called from whole genome sequencing using Trost et al., published pipeline [43].

CNVs were annotated using ANNOVAR and the UCSC Genome Browser to map segmental duplications, centromeric, and HLA regions. We annotated genes encompassed in the CNVs using Gencode V19 annotation (the reference release for hg19 Human genome release) with ENSEMBL(https://grch37.ensembl.org/index.html).

Rare CNVs in each cohort were defined by: a) *frequency* <1/1000 in Data Genome Variants (DGV, hg19, http://www.dgv.tcag.ca) with an overlap of 70%; and b) the rare CNV is contained in <50% of regions that are present at >1% in DGV (i.e., which represent common variants).

We selected CNVs ≥50Kb; with less that 50% overlap with segmental duplications, centromeric or HLA regions. We only analyzed autosomal CNVs since the effect of gene dosage is not comparable between sex-linked and autosomal CNVs.

For harmonization we selected CNVs encompassing at least ten probes for all array technologies used across all cohorts.

### Sleep risk gene lists

*Circadian pathway* genes (*n* = 312) were identified in three genetic databases: KEGG Pathway (hsa04710/hsa04713) [44], AmiGO2(GO:0007623) [45], and REACTOME (R-HSA-1368108/R-HSA-400253. Gene sets were extracted in May 2019 from the Broad Institute’s Molecular Signatures Database. In addition to core clock genes in the SCN, this list comprises of a larger network of genes modifying circadian expression.

*Insomnia risk* genes (*n* = 1053) were extracted from the two largest GWAS on insomnia [33, 34]. All identified genes were filtered out if they were non-protein coding based on published datasets from The Human Genome Organization [46].

High confidence ASD-risk genes were collected from the SFARI Gene database (category 1; extracted 2021) [47] to examine overlaps between ASD risk, circadian pathway and insomnia risk genes. Circadian pathway and insomnia risk genes collectively, are referred to as “sleep risk genes”.

### Scores of intolerance to haploinsufficiency and brain expression

All CNVs were scored based on the *“*loss-of-function observed/expected upper bound fraction” (LOEUF) values of genes encompassed in CNVs as previously published [48]. For each CNV, we computed the sum 1/LOEUF of all genes encompassed in that CNV, hence a high CNV score indicates a strong intolerance toward inactivation. The differential stability (DS) score [49] is a correlation-based metric that assesses the reproducibility of spatial patterns of gene expression in the brain. The score was transformed and ranges between 0 and 1, where a higher score indicates stable gene expression in specific brain regions. We computed the sum of DS scores of all genes encompassed in each CNV. Brain modules defined by Hawrylycz et al., 2012 [50], categorizing patterned gene expression in 13 brain regions, were used to characterize where in the brain circadian pathway and insomnia risk gene expression highly occur.

**Fig. 1:**
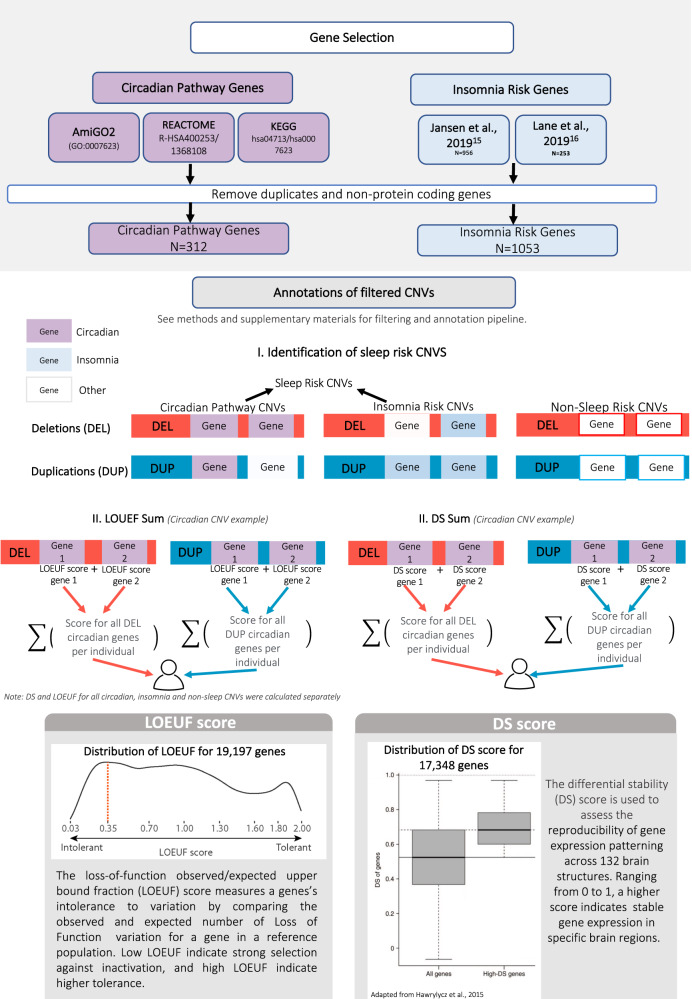
Schematic of CNV selection. The figure describes the selection and annotation process of CNVs included in the study. CNVs were first classified based on containing ‘sleep genes’ (circadian pathway or insomnia risk genes) or non-sleep genes. All genes encompassed within each CNV were then annotated to derive a sum of intolerance to haploinsufficiency (LOEUF score) and a brain expression score (DS Score).

Figure 1 provides a schematic of our workflow to identify sleep risk genes and annotate them with LOEUF and DS scores.

### Clinical and behavioral data

*The SSC Sleep Interview (SSCI)* is an 11-item parent report questionnaire assessing nighttime and daytime problems and sleep duration problems [51] see Supplemental Materials. Sleep data was only available for the SSC ASD probands. For the purposes of our study, we analyzed two sleep traits commonly reported as disturbed in ASD:

- Sleep duration: Average duration of sleep per week in minutes.
- Insomnia corresponding to two items: ‘*difficulty falling asleep’* and *‘frequent or prolonged awakenings at night*. These items parallel self-reports used to measure insomnia in Jansen et al., 2019 [33] and Lane et al., 2019 [34]. The number of insomnia traits was scored from 0 to 2. Completed responses for both items were required to compute the insomnia score.

Other binary sleep traits, including daytime sleepiness and troubles waking up in the morning were extracted for descriptive purposes.

*Cognitive ability*. Non-verbal IQ (NVIQ) data were available for the ASD cohorts and the IMAGEN dataset. A separate *g* factor score measuring general intelligence was available for GS. Cognitive assessment methods for each cohort are detailed in Supplementary Materials. No cognitive information was collected for unaffected siblings.

*The Autism Diagnostic Observation Scale calibrated severity score (ADOS CSS)* [52] is a 10-point scale based on raw ADOS scores. It captures overall ASD symptom severity independent of age and language level. Higher scores indicate greater symptom severity.

### Data analysis

All analyses were performed with R 3.6.3 (Supplementary Materials).

### Association between ASD and circadian and insomnia risk genes

Bayesian logistic regression analysis was used to estimate this association as follows:

$$\begin{array}{l}{{{\mathbf{Model1}}}}:{{{\mathrm{logit}}}}\left( {{{{\mathrm{ASD}}}}} \right) \sim \beta 0 + \beta 1,{}_{{{{\mathrm{DEL}}}}\left( {{\sum} {{{{\mathrm{sleep}}}}\,{{{\mathrm{risk}}}}\,{{{\mathrm{genes}}}}} } \right)} + \beta 2,{}_{{{{\mathrm{DUP}}}}\left( {{\sum} {{{{\mathrm{sleep}}}}\,{{{\mathrm{risk}}}}\,{{{\mathrm{genes}}}}} } \right)}\\\qquad\qquad\qquad\qquad\qquad\;\; +\, \beta 3,{}_{{{{\mathrm{DEL}}}}\left( {{\sum} {{{{\mathrm{other}}}}\,{{{\mathrm{non}}}} {\mbox{-}} {{{\mathrm{sleep}}}}\,{{{\mathrm{risk}}}}\,{{{\mathrm{genes}}}}} } \right)}\\\qquad\qquad\qquad\qquad\qquad\;\; +\,\beta 4,{}_{{{{\mathrm{DUP}}}}\left( {{\sum} {{{{\mathrm{other}}}}\,{{{\mathrm{non}}}} {\mbox{-}} {{{\mathrm{sleep}}}}\,{{{\mathrm{risk}}}}\,{{{\mathrm{genes}}}}} } \right)}\end{array}$$

$${\rm{DEL}} = {\rm{Deletion}};{\rm{DUP}} = {\rm{Duplication}}$$

where 0 is the intercept and 1–4 are the regression coefficients. Other non-sleep genes refer to protein-coding genes in the genome that are non-circadian pathway and non-insomnia risk genes, encompassed in a CNV.

*Secondary analyses*. Follow up logistic regression analyses investigating the effects of gene intolerance (1/LOEUF) and brain expression (DS_Score_)were also conducted.

$$\begin{array}{l}{{{\mathbf{Model2}}}}:{{{\mathrm{logit}}}}\left( {{{{\mathrm{ASD}}}}} \right) \sim \beta 0 + \beta 1,{}_{{{{\mathrm{DEL}}}}\left( {{\sum} {{{{\mathrm{sleep}}}}\,{{{\mathrm{risk}}}}\,{{{\mathrm{genes}}}}} } \right)} \times {\sum} {\left( {1/{{{\mathrm{LOEUF}}}}} \right)}\\\qquad\qquad\qquad\qquad\qquad\;\; +\, \beta 2,_{{{{\mathrm{DUP}}}}\left( {{\sum} {{{{\mathrm{sleep}}}}\,{{{\mathrm{risk}}}}\,{{{\mathrm{genes}}}}} } \right)} \times {\sum} {\left( {1/{{{\mathrm{LOEUF}}}}} \right)}\\\qquad\qquad\qquad\qquad\qquad\;\; +\,\beta 3,{}_{{{{\mathrm{DEL}}}}\left( {{\sum} {{{{\mathrm{other}}}}\,{{{\mathrm{non}}}} {\mbox{-}} {{{\mathrm{sleep}}}}\,{{{\mathrm{risk}}}}\,{{{\mathrm{genes}}}}} } \right)} \times {\sum} {\left. {1/{{{\mathrm{LOEUF}}}}} \right)} \\\qquad\qquad\qquad\qquad\qquad\;\; +\,\beta 4,_{{{{\mathrm{DUP}}}}\left( {{\sum} {{{{\mathrm{other}}}}\,{{{\mathrm{non}}}} {\mbox{-}} {{{\mathrm{sleep}}}}\,{{{\mathrm{risk}}}}\,{{{\mathrm{genes}}}}} } \right)} \times {\sum} {\left( {1/{{{\mathrm{LOEUF}}}}} \right)} \end{array}$$

1/LOUEF scores per gene was summed by type of CNV (DEL/DUP) and gene category (sleep/non-sleep risk genes). This same model was used with DS scores.

For all models above, two additional identical models were performed separating CNV’s encompassing circadian and insomnia genes. Models comparing ASD probands and unaffected siblings are adjusted for familial relationship with a random effect. Follow up models above were controlled for cognitive ability.

*Bootstrapping*. To test how robustly CNVs encompassing selected sleep risk genes better-predicted autism risk, we compared 95% confidence intervals obtained using a bootstrap procedure (1000 iterations) between sleep and non-sleep gene duplications and deletions. Confidence intervals reported are the mean of aggregated bootstrap results.

### Associations between sleep genes and SSC sleep phenotype

A linear regression was applied to test the association between sleep duration in minutes and sleep risk genes encompassed in deletions of duplications.

$$\begin{array}{l} < {{{\mathbf{Model3}}}}:{{{\mathrm{Sleep}}}}\,{{{\mathrm{Duration}}}}\left( {{{{\mathrm{minutes}}}}} \right) \sim \beta 0 + \beta 1,\,_{{{{\mathrm{DEL}}}}\left( {{\sum} {{{{\mathrm{sleep}}}}\,{{{\mathrm{risk}}}}\,{{{\mathrm{genes}}}}} } \right)}\\\qquad\qquad\qquad\qquad\qquad\qquad\qquad\qquad\quad\;\;+\, \beta 2,\,_{{{{\mathrm{DUP}}}}\left( {{\sum} {{{{\mathrm{sleep}}}}\,{{{\mathrm{risk}}}}\,{{{\mathrm{genes}}}}} } \right)}\\\qquad\qquad\qquad\qquad\qquad\qquad\qquad\qquad\quad\;\;+\,\beta 3,\,_{{{{\mathrm{DEL}}}}\left( {{\sum} {{{{\mathrm{other}}}}\,{{{\mathrm{non}}}} {\mbox{-}} {{{\mathrm{sleep}}}}\,{{{\mathrm{risk}}}}\,{{{\mathrm{genes}}}}} } \right)}\\\qquad\qquad\qquad\qquad\qquad\qquad\qquad\qquad\quad\;\;+\, \beta 4,\,_{{{{\mathrm{DUP}}}}\left( {{\sum} {{{{\mathrm{other}}}}\,{{{\mathrm{non}}}} {\mbox{-}} {{{\mathrm{sleep}}}}\,{{{\mathrm{risk}}}}\,{{{\mathrm{genes}}}}} } \right)}\end{array}$$

An ordinal logistic regression was used to analyze the association between sleep risk genes and the number of insomnia traits (three levels: 0, 1, 2).

$$\begin{array}{l} < {{{\mathbf{Model4}}}}:{{{\mathrm{logit}}}}\left( {{{{\mathrm{Insomnia}}}}\,{{{\mathrm{traits}}}}} \right) \sim \beta 0 + \beta 1,\,_{{{{\mathrm{DEL}}}}\left( {{\sum} {{{{\mathrm{sleep}}}}\,{{{\mathrm{risk}}}}\,{{{\mathrm{genes}}}}} } \right)}\\\qquad\qquad\qquad\qquad\qquad\qquad\qquad\quad\;\,\;\;+\, \beta 2,\,_{{{{\mathrm{DUP}}}}\left( {{\sum} {{{{\mathrm{sleep}}}}\,{{{\mathrm{risk}}}}\,{{{\mathrm{genes}}}}} } \right)}\\\qquad\qquad\qquad\qquad\qquad\qquad\qquad\quad\;\,\;\; +\, \beta 3,\,_{{{{\mathrm{DEL}}}}\left( {{\sum} {{{{\mathrm{other}}}}\,{{{\mathrm{non}}}} {\mbox{-}} {{{\mathrm{sleep}}}}\,{{{\mathrm{risk}}}}\,{{{\mathrm{genes}}}}} } \right)}\\\qquad\qquad\qquad\qquad\qquad\qquad\qquad\quad\;\,\;\;+\, \beta 4,\,_{{{{\mathrm{DUP}}}}\left( {{\sum} {{{{\mathrm{other}}}}\,{{{\mathrm{non}}}} {\mbox{-}} {{{\mathrm{sleep}}}}\,{{{\mathrm{risk}}}}\,{{{\mathrm{genes}}}}} } \right)}\end{array}$$

Relevant co-variates were added to all sleep phenotype models above (Supplementary Materials). Additional analyses to determine the association between sleep phenotypes and the sum of 1/LOEUF and DS_score_ per CNVs were conducted. All models were followed up by separating CNV’s encompassing circadian and insomnia genes.

### Multiple comparisons

Bonferroni corrections were applied for each main model to account for our three genetic constraint scores tested (i.e., binary,1/LOEUF, DS_score_). Significance was corrected to *p* = 0.017, subsequent sensitivity analyses were not corrected for.

## Results

### Associations between sleep (circadian and insomnia) risk genes and autism risk

**Fig. 2:**
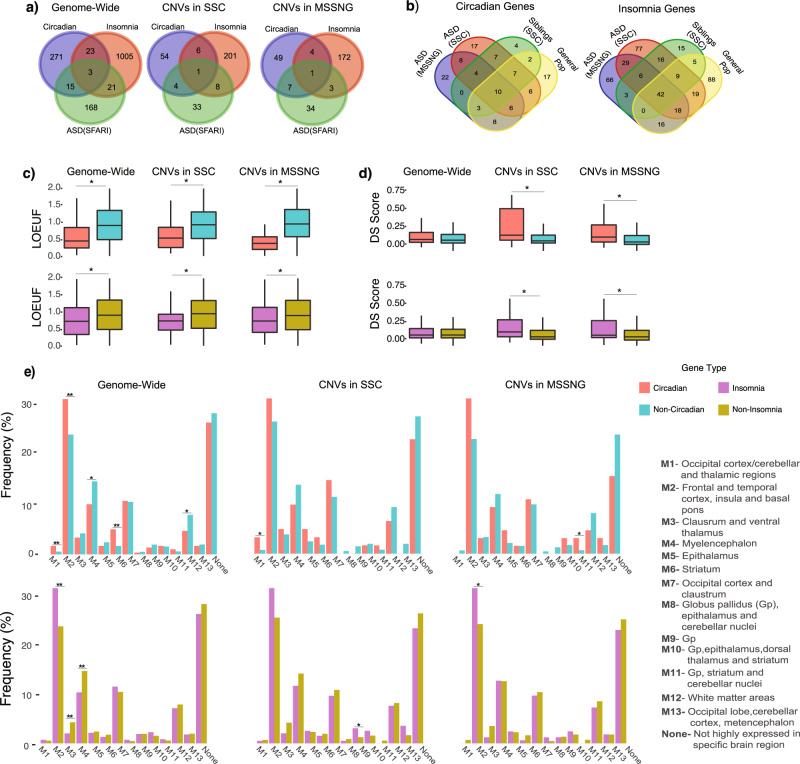
Sleep risk gene characteristics. **a** Overlaps between insomnia risk, circadian pathway and ASD risk genes are depicted across the genome and for genes identified in SSC and MSSNG ASD cohorts. **b** Overlap between insomnia risk and circadian pathway genes encompassed in CNV’s in each cohort. **c** LOEUF score comparisons between insomnia risk and circadian pathway genes are depicted across the genome and for genes identified in SSC and MSSNG ASD cohorts. Mann–Whitney-U tests were applied for statistical comparisons. **d** DS score comparisons between insomnia risk and circadian pathway genes are depicted across the genome and for genes identified in SSC and MSSNG ASD cohorts. Mann–Whitney-U tests were applied for statistical comparisons. **e** Brain module comparisons between insomnia risk and circadian pathway genes are depicted across the genome and for genes identified in SSC and MSSNG ASD cohorts. Modules provide the locations brain regions where gene expression is concentrated (see legend). Binomial linear regressions were used to compare modules and FDR corrections were applied.

Demographic data is found in Table 1. Overall, we identified 263 duplications and 72 deletions encompassing circadian pathway genes, as well as 362 duplications and 254 deletions encompassing insomnia risk genes (Table 1). There was minimal overlap between our list of sleep risk genes and the SFARI list of high confidence ASD risk genes (Fig. 2a). The overlap of circadian and insomnia genes across cohorts are described in Fig. 2b and further characterized by gene name, chromosome location, inheritance, and frequency for all 5 cohorts in Tables S1–S5.

**Table 1:** Cohort descriptives.

Variables	General population	SSC ASD probands	MSSNG ASD probands	SSC siblings
Demographic characteristics				
^**a**^*N* individuals	7509	2571	3289	2092
^**b**^Mean age (SD)	42.0(19.36)	9.03 (3.57)	9.18 (4.39)	11.65 (3.76)
^**c**^Sex (male%)	41.7	86.7	78.8	16.3
^**d**^*N* available Cognitive Test	7225	2562	1397	n/a
^**e**^Mean Z score cognitive ability	0.12	−1.03	−0.47	n/a
ADOS overall	n/a	7.45	7.28	n/a
CNV frequency				
*N*(%) carriers of Circadian DUP	72(0.96)	72(2.80)	80(2.43)	36(1.72)
*N*(%) carriers of Non-Circadian DUP	1276(16.99)	702(27.30)	953(28.97)	525(25.09)
*N*(%) carriers of Circadian DEL	12(0.16)	31(1.20)	22(0.67)	7(0.33)
*N*(%) carriers of Non-Circadian DEL	669(9.30)	348(13.53)	443(13.47)	235(11.23)
*N*(%) carriers of Insomnia DUP	136(1.81)	99(3.85)	82(2.49)	45(2.15)
*N*(%) carriers of Non-Insomnia DUP	1240(16.51)	687(26.72)	947(28.79)	519(24.80)
*N*(%) carriers of Insomnia DEL	85(1.13)	71(2.76)	62(1.88)	36(1.72)
*N*(%) carriers of Non-Insomnia DEL	610(8.12)	315(12.25)	410(12.47)	211(10.09)

^a^Imagen (*N* = 1744); Generation Scotland (*N* = 5481).

^b^Imagen (Mean_age_ = 12.4, Mean_SD_ = 0.36); Generation Scotland (Mean_age_ = 50.7, Mean_SD_ = 13.28).

^c^Imagen (Male = 49%)); Generation Scotland (Male = 39.5%).

^d^Imagen (Ntests = 1744); Generation Scotland (Ntests = 5481).

^e^Imagen (0.44); Generation Scotland (0.02).

#### Deleting or duplicating sleep risk genes is associated with autism risk

**Fig. 3:**
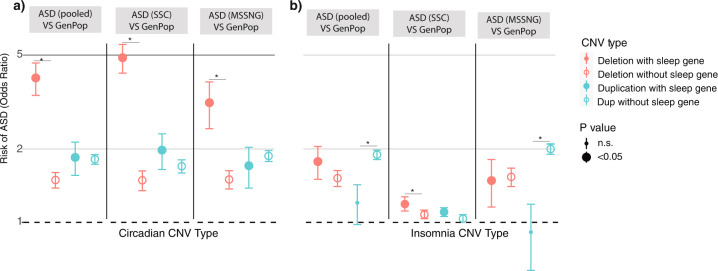
Associations between ASD and sleep risk genes. **a** Associations between ASD risk and CNVs encompassing circadian pathway genes. **b** Associations between ASD risk and CNVs encompassing insomnia risk genes. *Indicates bootstrap analyses show no overlapping 95% CI.

Pooling the MSSNG and SSC datasets yielded highly significant enrichment of insomnia risk (OR = 1.7, 95% CI = 1.5–2.0, *p* = 6.1^–05^) and circadian pathway deletions (OR = 4.0, 95% CI = 3.4–4.6, *p* = 1.3^–05^), and circadian duplications (OR = 1.8, 95% CI = 1.5–2.1, *p* = 4.4^–05^), in ASD compared to controls (see Fig. 3 and Table S6). 95% CI of these ORs obtained by bootstrapping confirmed these results (Table S6).

Results remained significant in each separate cohort (Fig. 3 and Table S6). Sensitivity analyses showed that results were unaffected by using either inter-familial (siblings) or extra-familial controls (Table S6). Additional analysis showed that deletions containing circadian, and, to a lesser extent, insomnia risk genes were better predictors of ASD risk compared to deletions encompassing other genes (Table S6). Effects of circadian genes and insomnia-risk genes remained significant after controlling for IQ (Table S7). Excluding all recurrent neuropsychiatric CNVs [42] demonstrated similar enrichments of circadian and insomnia-risk genes in the pooled dataset (Table S6). To disentangle if sleep risk gene enrichments resulted from the known excess of large CNVs in ASD compared to controls, we performed a series of stringent sensitivity analyses (see supplementary methods). Circadian and insomnia genes remained enriched in CNVs observed in the ASD group when we compared them to randomly sampled (genome wide) CNVs matched for the number of genes and level of intolerance to haploinsufficiency observed in the ASD cohorts (Figs. S1, S2).

#### Insomnia-risk genes intolerant to haploinsufficiency increase ASD risk

Based on previous studies [36], it was expected that autism risk uncovered above would be driven by genes intolerant to haploinsufficiency. Circadian and insomnia genes showed a distribution of LOEUF values significantly skewed towards intolerance (Fig. 2c). For insomnia genes, ASD risk was associated with increasing intolerance to haploinsufficiency measured by LOEUF in the pooled dataset for duplicated (β = 0.06, *p* = 1.7^–03^) and deleted (*β* = 0.17, *p* = 3.4^–05^) insomnia genes compared to the general population and when compared with siblings (Table S8). However, measures of intolerance did not influence ASD risk conferred by circadian genes (Table S8).

#### Brain expression of sleep genes is associated with autism risk

We expected that circadian and insomnia risk genes with stronger patterned brain expression (measured by DS score [49]) would be related to autism risk. The full list of circadian genes show a slightly higher DS score compared to non-circadian genes (Fig. 2d) and were concentrated in three brain modules, including thalamic regions involved in sleep regulation (Fig. 2e). Insomnia genes were also enriched in three brain modules, but not in primary brain regions (e.g., hypothalamus) regulating sleep (Fig. 2e). However circadian and insomnia risk genes encompassed in CNVs identified in both ASD cohorts had a slightly higher DS score compared to non-sleep genes (Fig. 2d).

In both cohorts, ASD risk was related to higher DS scores for circadian (*β* = 0.6, *p* = 1.8^–03^) and insomnia risk (*β* = 0.23, *p* = 4.1^–03^) genes encompassed in duplications but not deletions (Table S10). Only a higher DS of insomnia duplications were associated with ASD risk when compared to siblings (Table S9).

### Associations between CNVs and sleep phenotypes

#### Descriptives

**Fig. 4:**
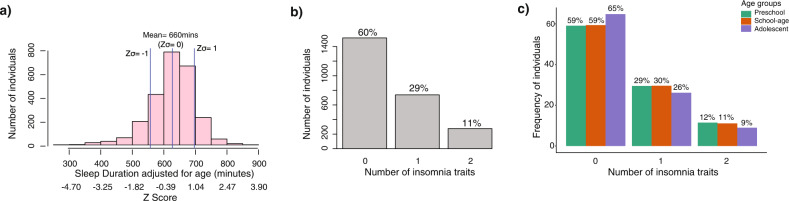
Sleep trait descriptions in the SSC ASD cohort. **a** Sleep duration distribution adjusted for age. **b** Frequency of insomnia traits. **c** Frequency of insomnia traits in each developmental age group.

Sleep duration and insomnia traits were available for 2473 and 2532 SSC probands respectively. School-aged children (5–12 years) made up the majority of the cohort (*N* = 1647), followed by preschoolers aged 4–5 years (*N* = 584), then adolescents aged 13–18 years (*N* = 301). Mean sleep duration adjusted for age was 660 min (Fig. 4). Age and NVIQ, were weakly associated with sleep duration, while sex and ADOS overall severity were not (Table S10). 39% of individuals in SSC had at least one insomnia trait (Fig. 4). The adolescent group had fewer insomnia traits (~35%), compared to school age and preschool children (~40%; Fig. 4). NVIQ and sex (i.e., females had more insomnia) were the only covariates associated with insomnia traits-albeit weakly (Table S11). Probands with two insomnia traits were twice as likely to experience daytime sleepiness (37%) and difficulties waking up in the morning (29%) compared to the rest of the cohort (14 and 17% respectively; Table S12). These associations demonstrate the negative impact insomnia traits have on daily functioning.

#### Circadian and Insomnia risk CNVs were not associated with sleep duration

Association analyses investigating circadian pathway duplications and deletions separately revealed ASD youth with a rare circadian gene duplication showed a decrease of 19.1 min (−0.25 z-score) per weeknight compared to ASD youth without such duplications (*p* = 0.03) (Table S13), but this did not pass Bonferroni corrections. Furthermore, we did not observe any differences in parental reports of daytime sleepiness or difficulties waking up in the morning in the group with circadian duplications compared to non-carriers. Similarly, insomnia risk CNVs were not associated with sleep duration in the ASD SSC cohorts (Table S13).

In contrast, deletions (but not duplications) encompassing non-circadian genes scored by LOEUF or DS, led to an increase in sleep duration (Table S13).

#### Insomnia genes have mild effects on insomnia traits

The presence of a CNV encompassing a circadian or insomnia risk gene was not associated with an insomnia trait. However, insomnia genes measured by LOEUF scores (measuring intolerance to haploinsufficiency) were associated with increasing the likelihood of having insomnia traits when duplicated (albeit with a small effect, OR = 1.05, 95% CI = 1.01–1.09, *p* = 1.2^–03^) (Table S14). When individuals with psychiatric CNVs were removed, these findings became marginally significant (*p* = 0.05). Stratification by age revealed that these results were driven by school-age participants (OR:1.05, 95% CI = 1.01–1.09, *p* = 1.4^–02^), who make up the majority of the cohort.

Conversely, duplications encompassing non-insomnia and non-circadian genes were associated with fewer insomnia traits. The DS score of sleep risk and non-sleep risk genes was not significantly associated with insomnia traits (Table S14).

## Discussion

We investigated the effects of CNVs encompassing circadian and insomnia risk genes on ASD risk and sleep traits. We show that circadian pathway genes increase ASD liability even after adjusting for cognitive abilities, these findings were less robust for insomnia CNVs. Circadian pathway deletions were better indicators of ASD risk compared to deletions that did not contain circadian genes, without influencing sleep phenotypes. Insomnia risk genes were associated with insomnia traits without altering sleep duration. Frequently identified circadian and insomnia risk CNVs in both ASD cohorts included well known recurrent CNVs linked to ASD susceptibility and sleep problems, such as 16p11.2 and 15q11.2–13.1 [53–55]. However, sensitivity analyses removing recurrent CNVs yielded the same enrichment of CNVs encompassing circadian and insomnia risk genes in ASD. Our results suggest no one specific CNV is responsible for these enrichments.

### Circadian pathway genes increase ASD risk without observable impacts on sleep traits

It is well established that dysregulation of circadian rhythms (e.g., shift workers, sleep phase disorders) is linked with a host of psychiatric disorders, medical conditions, and cognitive and behavioral impairments [56]. However, candidate SNP studies are unable to provide robust evidence of associations between circadian clock genes and psychiatric disorders [57, 58].

Our findings provide evidence for the contribution of circadian pathway dysfunction to ASD, but the mechanisms by which these genes increase ASD risk are unknown. Previous theories have put forth sleep disturbance as a causal factor for ASD risk and other psychopathologies. These theories draw on evidence from mouse models indicating that sleep disturbances early in life can lead to alterations of plasticity, brain maturation, and organization [21]. However, in this study, the limited effects of circadian genes on sleep duration and insomnia are unlikely to disrupt behavior and cognition enough to increase ASD risk. Instead, we propose that circadian pathway genes increase ASD risk with minimal to no modulation of sleep duration and insomnia. Alternatively, it is also possible that these circadian gene mutations are responsible for significantly disturbing sleep at the electrophysiological level, which may not be captured by parent reports. Such discordance between atypical electrophysiological sleep and normal sleep reported by parents in youth with ASD has previously been reported [59]. Further investigations including objective physiological sleep measures are warranted. Moreover, mouse models of ASD provide a promising approach to understanding genetic mechanisms underlying the link between sleep regulation, circadian rhythms, and ASD [60, 61]. For instance, mouse models of ASD candidate gene Shank3 were found to have insomnia-related difficulties (i.e., long sleep onset), but no circadian abnormalities [32]. This is line with observations in human GWAS suggesting distinct biological mechanism are related to different sleep traits, with little association to circadian pathway genes. For instance, modest overlaps between insomnia genetic loci and other sleep traits, like duration and chronotype, have previously been found [33]. As we demonstrate, genes within these loci regions also minimally overlap with circadian pathway genes.

#### Insomnia risk genes increase ASD and insomnia risk

Contrary to the largest insomnia GWAS [33] showing no genetic correlation between insomnia and ASD, we found that the gene list provided by both GWAS studies increased ASD liability when deleted. Although insomnia risk genes within CNVs identified in ASD cohorts showed higher patterned expression in the brain and slightly increased intolerance to haploinsufficiency compared to other coding genes in the genome, neither characteristic was clearly responsible for driving the association between insomnia risk genes and ASD. The genetic correlations between insomnia and multiple psychiatric symptoms and conditions suggest that underlying genes target mechanisms responsible for psychopathology, whereby poor sleep is one among many symptoms. As an example, the 16p11.2 deletion, which encompasses insomnia risk genes, is known to increase ASD risk and studies in mice and humans have observed circadian rhythm and sleep disturbances in those carrying this deletion [53, 54]. Strengthening this theory, our study demonstrates that insomnia risk genes are not highly expressed in primary sleep regulation centers (i.e., hypothalamus), but rather other areas in the brain.

#### Parent reported sleep disturbances were not over represented in the SSC cohort

Proponents of the circadian dysfunction theory suggest disturbances of circadian sleep rhythmicity may increase the vulnerability of developing ASD symptomology [21, 27]; our study did not support this. Rather, we showed that sleep problems of youth with ASD in the SSC cohort were comparable to what has previously been reported in typically developing cohorts [62, 63]. Only 4% of SSC youth slept less than the National Sleep Foundation’s recommended guidelines for their age [63]. A previous SSC study reported a higher rate (~25%) of youth that did not meet recommended sleep duration, but classified those with “may be acceptable” duration as poor sleepers [51].

Although insomnia traits in SSC were linked to greater daytime sleep consequences, reports of two insomnia traits in the SSC cohort (10%) were drastically lower than reports in typically developing youth populations [64]. These comparisons should be interpreted with caution given that insomnia traits in SSC lack information about their severity and frequency. Moreover, ASD severity, and specifiers such as NVIQ, had almost no association with sleep duration or insomnia traits. Hence, previous suggestions that sleep problems in ASD may occur from an ineffectiveness to process *environmental cues* that entrain circadian rhythms due to social and communication difficulties, were not observed in the SSC.

### Limitations

Investigating rare variants affecting gene lists representing less than 10% of the coding genome requires powerful datasets. In particular there are less than 25 known core clock genes. Slightly different results between ASD cohorts may be due to power issues and noise introduced by different technologies (microarray vs whole genome sequencing), which may contribute to discrepancies in CNV identification.

Given the limited sleep phenotypes, and in particular the absence of phenotypes in unaffected siblings and controls, we were unable to establish the normative association between sleep phenotypes and our genes of interest. Moreover, the current sleep questionnaire used in the SSC cohort is not validated nor does it conform to accepted field standards put forth to standardize the evaluation of health-related outcomes in research and clinical practice [65–67]. Hence sleep phenotypes evaluated by the SSCI, including the lack of disturbance found in the SSC cohort, should be interpreted with caution. Objective sleep measures are needed to validate findings between rare gene variants and sleep problems in ASD. Larger sample sizes are also needed to investigate the effects between gene variants and sleep traits during distinct developmental periods, as sleep and circadian rhythms change across childhood [68]. We were not able to adjust for medication impacting sleep, which is commonly administered to ASD youth. Specifically, the use of melatonin known to ameliorate sleep problems in ASD [30], was not documented in SSC.

## Conclusion

Our results implicate rare circadian and insomnia risk gene variants with increase likelihood of ASD risk and minimal impact on sleep traits, suggesting pleiotropic effects for these genes [16]. We are currently unable to compare our findings to studies of a similar or larger scale, hence further investigations in health and disease are required to delineate the phenotypic effects of circadian pathways and insomnia risk genes. Future studies investigating the combined effect of genomic variants and environmental factors on sleep measures, behavioral traits, and brain architecture are needed for a holistic understanding of the interplay between genes, sleep and ASD.

## Supplementary information

SUPPLEMENTAL TABLES

SUPPLEMENTAL MATERIALS
