# Supplementary material for: Investigating the contributions of circadian pathway and insomnia risk genes to autism and sleep disturbances
Source: Transl Psychiatry. 2022 Oct 3;12:424. doi: 10.1038/s41398-022-02188-2 (PMC9529939; doi:10.1038/s41398-022-02188-2)
Supplement: Supplementary file 2 — SUPPLEMENTAL MATERIALS [file 41398_2022_2188_MOESM2_ESM.docx]

**SUPPLEMENTARY MATERIALS ONLINE CONTENT**

**METHODS**

**Genetics data**

CNV Technology

- SSC: *Microarray (*1Mv1, 1Mv3, and Omni 2.5)
- MSSNG: *Whole Genome Sequencing (*HiSeq, HiSeq 2,500, and HiSeqX)
- IMAGEN: *Microarray (*Illumina Quad 610 / 660 chips)
- Generation Scotland: *Microarray (*Illumina GSA)

CNV Detection

CNV detection algorithms from the software PennCNV[1] and QuantiSNP[2] , were used with

the following parameters: a) 3 consecutive probes for CNV detection; b) CNVs > 1 Kb; and c)

confidence scores ≥ 15. They were then combined with CNVision[3] . After this merging step,

the CNV Inheritance Analysis algorithm (developed by the Thomas Bourgeron’ lab), was applied

to concatenate adjacent CNVs into one, according to the following criteria:  a) gap

≤150 Kb between the CNVs; b) size of the CNVs ≥ 1000 bp; and c) number of probes ≥ 3. Visual

validations were performed with SnipPeep (<http://snippeep.sourceforge.net/)>. Finally, random

forest validation was used to filter CNVs with an algorithm trained on ~ 24,000 CNV

visualizations.

*De novo* CNVs in the SSC were identified in probands, unaffected siblings, and unselected populations using two previously published datasets[3, 4], combined with our own algorithm developed in R [5]. A CNV was considered as *de novo* only if it was defined as such by all three approaches.

*Psychiatric CNVs.* Recurrent psychiatric CNVs removed for sensitivity analyses were accessed from Huguet et al., 2018[6].

**Clinical and behavioral data**

Cognition

*SSC Autism Cohort. N*on-verbal intelligence quotient (NVIQ) scores were obtained from the Differential Ability Scales, 2nd Edition (DAS-II)[7] for early years (N=1,030) and school age children (N=1,212), the Wechsler Intelligence Scale for Children, 4th Edition (WISC-IV) (27) (N=45), the Wechsler Abbreviated Scale of Intelligence – First Edition (WASI-I)[8] (N=62) or the Mullen Scales of Early Learning (MSEL)[9] (N=213). Norm-referenced standard scores (deviation NVIQ) were available for most of the participants. However, for individuals from SSC who were not able to obtain a deviation NVIQ due to their age and/or developmental level, ratio IQ were derived by dividing mental age by chronological age and multiplying by 100. See Bishop et al., 2011 for more details concerning convergence between ratio and deviation NVIQ[10].

*MSSNG Autism Cohort.* NVIQ scores were obtained from the Leiter international performance scale – Original and revised[11,12](N=372), the raven progressive matrices[13](N=214), the Stanford-Binet intelligence scale (N=281), the Wechsler Intelligence Scale for Children – Fourth Edition (WISC-IV)[14] (N=46), the Wechsler Abbreviated Scale of Intelligence – First and Second Editions (WASI-I, WASI-II) (N=338) or the Wechsler Preschool and Primary Scale of Intelligence – Fourth Edition (WPPSI-IV) (N=128).

In ASD cohorts, we did not adjust for age as we adjusted for the type of test used for assessing IQ that already takes into account the age (as well as language level) of the individual. Cognitive scores were derived as follows:

Linear Regression = glm(Cognitive ability ZScore ~ Cognitive Test Type, data= ASD Cohort)
ASD Cohort$residual_Zscore = residuals(Linear Regression)

*Imagen Cohort.* Scores were obtained from the fourth edition of the Wechsler intelligence scale for children (WISC-IV). Deviation NVIQ were available for all participants.

*Generation Scotland Cohort.* The g-factor is based on four cognitive tests measuring processing speed, verbal declarative memory, executive functions and vocabulary. The g-factor represents 42.3% of the observed variance. The g-factor was then transformed to a z-score using the mean of -3.649 x **~**10^-16^ and the SD of 1.3. For more details see Huguet et al., 2021 [15]

Sleep

To date four SSC papers have been published using the Sleep Interview (SSCI), to examine parent reported sleep disturbances in relation to behavioral, medical and cognitive phenotypes, and most recently with common genetic variants[16–19]. Within the SSC cohort, nighttime problems, particularly difficulty going to sleep, are the most frequently reported sleep issue[17]. These nighttime scores have been associated with medical and cognitive problems, like gastrointestinal issues and lower NVIQ. To date, items on the SSCI have been aggregated into subscales and global composite scores, while specific sleep phenotype items have yet to be explored in depth. Unlike previous SSC sleep characterizations, current analyses will be more specific to sleep traits related to insomnia that are reported to be elevated in the ASD population.

**DATA ANALYSIS**

| **Regression model** | **Pagckage** | **Function** | **Other** |
| --- | --- | --- | --- |
| Bayesian logistic regression | ‘*arm’* | *bayesglm()* | random effect (1\| FID) **Control for family relations* |
| Ordinal logistic regression | *‘MASS’* | *Polr()* |  |
| Linear regression | ‘stats’ | *lm()* |  |

*R 3.6.3 Statistical models packages used:*

**SENSITIVITY ANALYSIS**

**Description of circadian pathway and insomnia risk gene distributions based on the number of genes within CNVs**

To remove uncertainty about whether an overrepresentation of CNVs encompassing circadian and insomnia genes in ASD cohorts resulted from an overall excess of CNVs (hence more genes) compared to the general population, we compared ASD-CNVs with stochastically simulated CNVs. Random CNVs were created by selecting segments of the genome matching the number of contiguous genes within ASD-CNVs (weighted by gene distribution across chromosomes). In addition, we applied a haploinsufficiency constraint based on the pLI (probability of being Loss-of-function Intolerant) to create control-CNVs with a similar burden (± 10%). This stochastic set of CNVs were simulated 10,000 times to create a null distribution. Compared to this distribution, both circadian rhythm (Figure 1) and insomnia (Figure 2) genes are enriched in ASD-CNVs with p-values equal to 7$.7\times{10}^{-3}$ and $1\times{10}^{-4}$ respectively.

**Figure S1**


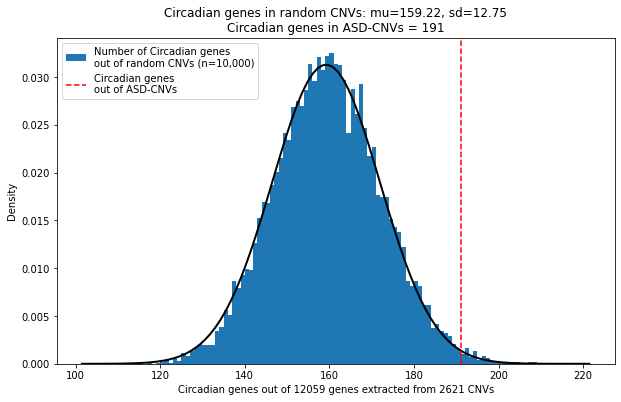
**Figure S2**


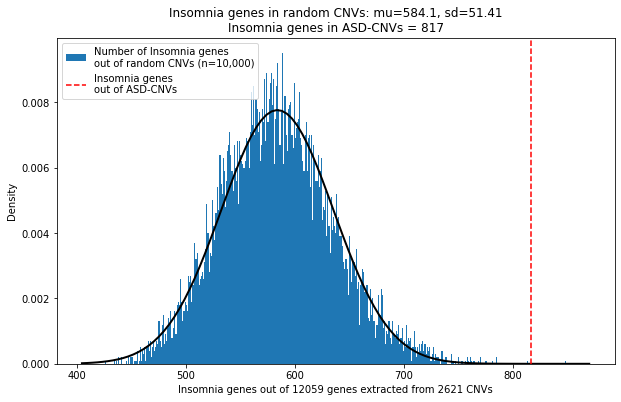


**References**

1. Wang Q, Chen R, Cao Z, Yuan H, Zhao W, Li X, et al. [Effects of simultaneous multi-level surgery intervention on the changes of the oxidative stress and the cognitive function in young and middle-aged patients with Moderate-severe obstructive sleep apnea hypopnea syndrome]. Lin Chung Er Bi Yan Hou Tou Jing Wai Ke Za Zhi. 2015;29:2139–2142.

2. Colella S, Yau C, Taylor JM, Mirza G, Butler H, Clouston P, et al. QuantiSNP: an Objective Bayes Hidden-Markov Model to detect and accurately map copy number variation using SNP genotyping data. Nucleic Acids Res. 2007;35:2013–2025.

3. Sanders SJ, Ercan-Sencicek AG, Hus V, Luo R, Murtha MT, Moreno-De-Luca D, et al. Multiple recurrent de novo CNVs, including duplications of the 7q11.23 Williams syndrome region, are strongly associated with autism. Neuron. 2011;70:863–885.

4. Krumm N, Turner TN, Baker C, Vives L, Mohajeri K, Witherspoon K, et al. Excess of rare, inherited truncating mutations in autism. Nature Genetics. 2015;47:582–588.

5. Douard E, Zeribi A, Schramm C, Tamer P, Loum MA, Nowak S, et al. Effect Sizes of Deletions and Duplications on Autism Risk Across the Genome. AJP. 2021;178:87–98.

6. Huguet G, Schramm C, Douard E, Jiang L, Labbe A, Tihy F, et al. Measuring and Estimating the Effect Sizes of Copy Number Variants on General Intelligence in Community-Based Samples. JAMA Psychiatry. 2018;75:447–457.

7. Elliott CD, Salerno JD, Dumont R, Willis JO. The Differential Ability Scales—Second Edition. Contemporary intellectual assessment: Theories, tests, and issues, 4th ed, New York, NY, US: The Guilford Press; 2018. p. 360–382.

8. Wechsler D. WASI-I: Wechsler abbreviated scale of intelligence. 1999.

9. Mullen EM. Mullen scales of early learning. Circle Pines, MN: AGS; 1995.

10. Bishop SL, Guthrie W, Coffing M, Lord C. Convergent Validity of the Mullen Scales of Early Learning and the Differential Ability Scales in Children With Autism Spectrum Disorders. American Journal on Intellectual and Developmental Disabilities. 2011;116:331–343.

11. Leiter R. Leiter international performance scale1. 1979.

12. Roid GH, Miller LJ. Leiter international performance scale-revised (Leiter-R). Stoelting. Wood Dale, IL; 1997.

13. Raven J, Court J, Raven J. Manual for Raven’s progressive matrices and vocabulary scales. 1998.

14. Wechsler D. Wechsler Intelligence Scale for Childrenn - Fourth Edition. 2003.

15. Huguet G, Schramm C, Douard E, Tamer P, Main A, Monin P, et al. Genome-wide analysis of gene dosage in 24,092 individuals estimates that 10,000 genes modulate cognitive ability. Mol Psychiatry. 2021:1–14.

16. Veatch OJ, Sutcliffe JS, Warren ZE, Keenan BT, Potter MH, Malow BA. Shorter sleep duration is associated with social impairment and comorbidities in ASD: Sleep duration relates to social impairment. Autism Research. 2017;10:1221–1238.

17. Johansson AEE, Feeley CA, Dorman JS, Chasens ER. Characteristics of sleep in children with autism spectrum disorders from the Simons Simplex Collection. Research in Autism Spectrum Disorders. 2018;53:18–30.

18. Johansson AEE, Dorman JS, Chasens ER, Feeley CA, Devlin B. Variations in Genes Related to Sleep Patterns in Children With Autism Spectrum Disorder. Biol Res Nurs. 2019;21:335–342.

19. Aldinger KA, Lane CJ, Veenstra-VanderWeele J, Levitt P. Patterns of Risk for Multiple Co-Occurring Medical Conditions Replicate Across Distinct Cohorts of Children with Autism Spectrum Disorder: Medical comorbidity patterns in autism. Autism Research. 2015;8:771–781.
